# Enhancing Solubility and Reducing Thermal Aggregation in Pea Proteins through Protein Glutaminase-Mediated Deamidation

**DOI:** 10.3390/foods12224130

**Published:** 2023-11-15

**Authors:** Lijuan Luo, Yuanyuan Deng, Guang Liu, Pengfei Zhou, Zhihao Zhao, Ping Li, Mingwei Zhang

**Affiliations:** 1 Sericultural & Agri-Food Research Institute Guangdong Academy of Agricultural Sciences, Key Laboratory of Functional Foods, Ministry of Agriculture and Rural Affairs, Guangdong Key Laboratory of Agricultural Products Processing, Guangzhou 510610, China; 2020309010032@webmail.hzau.edu.cn (L.L.);; 2 College of Food Science and Technology, Huazhong Agricultural University, Wuhan 430070, China; 3 Food Laboratory of Zhongyuan, Luohe 462300, China

**Keywords:** enzymatic deamidation, secondary structure, tertiary structure, solubility, thermal stability

## Abstract

The limited solubility and stability of pea proteins hinder their utilization in liquid formulations. In this study, protein glutaminase (PG) was employed to modify pea protein isolates (PPIs) and obtain deamidated PPI with varying degrees of deamidation (DD, 10–25%). The solubility and thermal stability of these deamidated PPI samples were assessed, and a comprehensive analysis, including SDS-PAGE, zeta potential, FTIR, surface hydrophobicity, and intrinsic fluorescence, was conducted to elucidate the mechanism behind the improvement in their functional properties. The results reveal that PG modification greatly enhances the solubility and heat stability of PPI, with the most notable improvements observed at higher DD (>20%). PG modification increases the net charge of PPI, leading to the unfolding and extension of the protein structures, thus exposing more hydrophobic groups. These structural changes are particularly pronounced when DD exceeds 20%. This increased electrostatic repulsion between carboxyl groups would promote protein unfolding, enhancing interactions with water and hindering the aggregation of unfolded protein in the presence of salts at elevated temperatures (supported by high-performance size exclusion chromatography and transmission electron microscopy). Accordingly, PG-mediated deamidation shows promise in enhancing the functional properties of PPI.

## 1. Introduction

Pea proteins, derived from *Pisum sativum* L., have recently gained substantial attention in the food industry due to their low allergenicity and environmentally sustainable characteristics [1,2]. However, their use in liquid food formulations is limited by poor water solubility and heat stability, primarily due to the prevalence of β-sheet structures within pea proteins [3,4]. To address these limitations and enhance the functionality, various methods, including physical, chemical, and enzymatic approaches, have been explored. Deamidation, a process converting amide groups into carboxyl groups, has shown promise in improving pea protein properties [5,6]. Deamidation can be achieved through acid or enzymatic methods, with the enzymatic approach being favored for avoiding protein hydrolysis [7,8,9].

Protein glutaminase (PG), derived from the soil bacterium *Chryseobacterium proteolyticum* strain 9670 T, is a promising enzyme for enzymatic deamidation. It can efficiently convert glutamine residues in proteins into glutamic acid without causing protein hydrolysis [7]. PG has been employed to enhance the solubility and emulsifying properties of various plant proteins [10,11]. Previous studies, such as YONG et al., have investigated structural and property changes in wheat gluten proteins induced by PG deamidation. They observed improved solubility and emulsification properties, along with changes in the β-sheet structure content [10]. Similarly, Suppavorasatit et al., focused on soy proteins deamidated to varying degrees and found enhanced emulsification and foaming properties, accompanied by protein chain breakage and alterations in secondary structure [11]. However, their structural analysis was limited to proteins with high deamidation degrees, and limited research has delved into the comprehensive investigation of structural changes and the resulting impact on the thermal aggregation behavior of plant proteins, specifically pea proteins, induced by deamidation.

In this study, pea protein was chosen as a model to investigate the mechanism linking structural changes induced by PG to functional properties. Pea proteins were isolated from raw materials and then subjected to incubation with PG to achieve different deamidation degrees. A comprehensive analysis involving SDS-PAGE, zeta potential, FTIR, surface hydrophobicity, intrinsic fluorescence, solubility, turbidity, HPSEC, and TEM was conducted to elucidate the effect of PG deamidation on the structural changes, as well as the solubility and heat stability (resistance against thermal aggregation) of pea proteins. The present work aims to provide a theoretical basis for a deeper understanding of the impact of PG-mediated deamidation on plant protein structure and functionality.

## 2. Materials and Methods

### 2.1. Materials

Yellow pea seeds (*Pisum sativum* L.) were acquired from Yantai Shuangta Food Co., Ltd. (Yantai, Shandong, China). Protein glutaminase ‘Amano 500’ (Activity 634 U/g) was purchased from Amano Enzyme, Inc. (Nagoya, Japan). O-Phthalaldehyde (P108632) was sourced from Aladdin in China. The serine standard (54763-100 mg) and 8-Anilino-1-naphthalenesulfonic acid ammonium salt hydrate (ANS, 216909-5 g) were obtained from Sigma-Aldrich. The prestained color protein marker (10–180 kDa, P0068) was procured from Beyotime in China. All other chemicals were obtained from Sinopharm Chemical Reagent Co., Ltd. (Shanghai, China) and were of analytical reagent grade. Deionized water was used for all experiments.

### 2.2. Preparation of Pea Protein Isolates (PPI)

Peas were milled and defatted via constant stirring with four times their weight of ether at room temperature for 3 h. After that, the defatted pea flours were filtered using skim gauze and then dried at 40 °C overnight. After drying, the defatted pea flours were used for protein extraction. Subsequently, pea protein isolates (PPIs) were separated from defatted pea flour using the isoelectric precipitation method as described by Peng et al., (2016) [12]. Briefly, 500 g of defatted pea flour was dispersed in distilled water (1:10, *w*/*v*). The resulting suspension was maintained at pH 8.0 and stirred for 2 h at room temperature (25–28 °C), followed by centrifugation at 8000× *g* for 30 min at 4 °C. The supernatants were adjusted to pH 4.5 with 2 mol/L HCl, stored for 2 h at 4 °C, and then centrifuged at 5000× *g* for 20 min at 4 °C. The precipitate was washed twice with distilled water, dissolved in distilled water, and then adjusted to pH 7.0 using 2 mol/L NaOH. After centrifugation (5000× *g*, 20 min, 4 °C), the supernatants were dialyzed against distilled water for 48 h at 4 °C and subsequently lyophilized. The protein content of the samples was determined to be 86.60% (*w*/*w*) on a dry basis, using the Kjeldahl method with a nitrogen conversion factor of 5.40 [12].

### 2.3. Preparation of Deamidated Pea Protein Samples

Deamidated pea protein isolates (DPPIs) were prepared using PG according to a modified method by Yong et al., (2006) [10]. Specifically, PPI was dissolved in 50 mM phosphate-buffered solution (PBS, pH 7.0) at a concentration of 20 mg/mL. It was then incubated in a water bath at 45 °C for 2 min. The PG (0.016 U/mg of protein) was added in the solution, and the reactions were carried out in sealed bottles at 45 °C for varying durations (0–24 h) to obtain DPPI samples with different deamidation levels. After the reaction, the sample solution was heated in a water bath at 80 °C for 20 min to inactivate the enzyme and then cooled in ice water bath. The amount of ammonia released after reaction was measured to determine the deamidation degree (DD), and curves plotting time against DD were generated. PPI treated with inactivated PG and subjected to the same incubation and high-temperature (80 °C) deactivation process was denoted as the heat control samples. 

Based on the obtained DD time curve, deamidated pea protein isolate samples with four different DDs (10%, 15%, 20% and 25%) were prepared through incubation at four distinct time intervals (3.91 min, 16.07 min, 65.97 min and 270.86 min, respectively) for further structural analysis. These resulting deamidated samples are designated as DPPI. PPI samples treated with inactivated PG enzyme for 65.97 min (similar to 20% DD) and then processed with high temperature (80 °C) were designed as heat control sample (denoted as HPPI). Subsequently, all samples were dialyzed against distilled water for 48 h at 4 °C and then subjected to lyophilization for further use.

### 2.4. Structural Characterization

#### 2.4.1. Determination of Released Ammonia (Degree of Deamidation)

The deamidation degree (DD) of PPI was calculated as the ratio of the net amount of released ammonia during the PG reaction to that from the total deamidation reaction. PPI (0.5 g) was totally deamidated via treatment with 2 mol/L HCl at 121 °C for 3 h. The amount of ammonia was determined using a Conway diffusion cell, following a previously described method [8]. Specifically, 3 mL aliquot of 2% boric acid solution containing methyl red and bromocresol green was added to the central chamber of a microdiffusion unit to capture the released ammonia gas. An aliquot (1 mL) of the sample solution was added to the outer chamber, where it mixed with preadded saturated K_2_CO_3_, and the cell was immediately sealed with a cover on the edges of coated gum Arabic. The Conway diffusion cell was put into an incubator at 30 °C for 12 h to release ammonia gas from the alkali sample solution. The ammonia absorbed in the 2% boric acid solution was titrated with 0.01 mol/L HCl.

#### 2.4.2. Determination of the Degree of Hydrolysis (DH)

The hydrolysis degree (DH) of deamidated pea protein isolate samples obtained at various reaction time intervals (0–24 h) was analyzed using the OPA method according to the method by Nielsen et al., (2001) [13]. Briefly, 7.620 g of borax and 200 mg of SDS were dissolved in 150 mL of deionized water to obtain a solution referred to as Solution A. Next, 160 mg of o-phthalaldehyde (OPA) was dissolved in 4 mL of anhydrous ethanol, and once completely dissolved, it was added to Solution A, followed by rinsing with deionized water and transfer. Then, 176 mg of 1,4-dithiothreitol was added to the above solution, followed by rinsing with deionized water and transfer. Finally, the resultant solution was brought to a final volume of 200 mL with deionized water to obtain the OPA reagent. A standard solution with serine-NH_2_ concentration of 0.9615 meqv/L was prepared by dissolving 10 mg of serine in 100 mL of deionized water. Briefly, 400 μL of the sample solution, serine standard solution, and deionized water were mixed in a test tube containing 3 mL of OPA reagent, mixed for 5 s, allowed to stand for 2 min, and the absorbance was immediately read at 340 nm. Each sample was repeated three times. The formula for calculating the degree of hydrolysis (DH) is as follows:

$$h=\frac{{\mathrm{serine}\;\mathrm{NH}}_{2}-\beta }{\alpha }\left[\frac{\mathrm{meqv}}{g\;\mathrm{protein}}\right]$$

$$\mathrm{DH}=\frac{h_{1}-h_{0}}{h_{\mathrm{tot}}}\times 100\%$$

where serine NH_2_ is the meqv serineNH_2_ per gram protein and h_0_ and h_1_ are the hydrolysis degree of the protein sample before and after deamidation, respectively. The constant values α, β and h_tot_ factor were 1.0, 4.0, and 8.0, respectively, based on theoretical general values for unexamined raw material [13].

#### 2.4.3. Sodium Dodecyl Sulfate-Polyacrylamide Gel Electrophoresis (SDS-PAGE)

An appropriate concentration of PPI samples dissolved in distilled water was mixed with four times the volume of 5× loading buffer containing β-mercaptoethanol. The mixture was then heated in boiling water for 5 min. The resulting loading solutions, each containing approximately 12 µg of sample, were loaded onto a sodium dodecyl sulfate-polyacrylamide gel consisting a 12% separating gel and a 5% stacking gel. A prestained protein ladder ranging from 10 to 180 kDa was loaded as a molecular weight marker. The obtained gel was stained with Comaisse^®^ blue stain solution for 60 min and subsequently destained with a solution containing 30% methanol, 10% acetic acid, and 60% water for 4 h. Finally, gel images were captured using a Bio-Rad image acquisition system (Bio-Rad Laboratories, Hercules, CA, USA).

#### 2.4.4. Zeta Potential Measurement

The proteins (DPPI, PPI, and HPPI) were dissolved in distilled water (0.5% *w*/*v*), and the pH of the resultant solution was adjusted to the desired values (pH 2–10) with either 2 mol/L NaOH or 2 mol/L HCl. Protein solution samples at different pH values were then subjected to zeta potential analysis at 25 °C using a Malvern Zetasizer Nano ZSE (Worcestershire, UK).

#### 2.4.5. Fourier Transform Infrared Spectroscopy (FTIR)

FTIR analysis of the DPPI samples was carried out using a Bruker Vertex 70 (Bruker, Mannheim, Germany). The samples were ground, mixed with KBr at a ratio of 1:100, and pressed to a pellet. The spectra were recorded in the range of 4000–400 cm^−1^ at an interval of 4 cm^−1^. The infrared spectrum of the amide-I region (1700–1600 cm^−1^) was deconvoluted to evaluate the secondary structures of the protein samples using Peakfit software (Version 4.12).

#### 2.4.6. Intrinsic Fluorescence Emission Spectroscopy

Fully hydrated protein dispersions (0.02%, *w*/*v*) were prepared in PBS (pH 7.0, 10 mmol/L) prior to intrinsic fluorescence analysis using a fluorescence spectrometer (F-7000; Hitachi, Tokyo, Japan). The fluorescence emission spectra were recorded from 300 nm to 500 nm with excitation at 290 nm (slits: at excitation, 10 nm; at emission: 5 nm).

#### 2.4.7. Surface Hydrophobicity (H_0_) Analysis

The H_0_ of the DPPI samples was determined using 8-(anilino)-1-naphthalene sulfonic acid ammonium salt (ANS), following the method described by Kato and Nakai, 1980 [14]. Protein solutions at various concentrations (0.004–0.02%, *w*/*v*) were prepared in PBS (pH 7.0, 10 mmol/L). An aliquot (10 µL) of ANS solution (8 mmol/L in 10 mmol/L PBS 7.0) was added to 1 mL of the protein solution. The relative fluorescence intensity of each sample was measured at an excitation wavelength of 390 nm and an emission wavelength of 470 nm using an Infinite M200pro microplate reader (Tecan, Mennedorf, Switzerland). A mixture without ANS (replaced by an equivalent volume of PBS) served as the blank. The initial slope of the fluorescence intensity plot as a function of protein concentration represented protein surface hydrophobicity (H_0_).

### 2.5. Determination of Solubility

The solubility of the DPPI samples was determined following the methods described by Adebiyi et al. with minor modifications [15]. The protein was dissolved in distilled water (0.5% *w*/*v*), and the pH of the resultant solution was adjusted to the desired values (pH 2–10) with either 2 mol/L NaOH or 2 mol/L HCl. A reference protein solution (0.5% *w*/*v*) was prepared in 0.1 M NaOH. After 60 min of stirring, all sample solutions were centrifuged at 10,000× *g* for 20 min. The protein content in the resultant supernatants was measured using Bradford’s method with bovine serum albumin as a standard. The protein solubility was calculated using the following equation:

Protein solubility (%) = (P_1_/P_2_) × 100

where P_1_ is the protein content in the supernatant of samples, and P_2_ is the protein content of the reference protein solution.

### 2.6. Thermal Properties

#### 2.6.1. The Turbidity (Heat Stability) of DPPI Solutions in the Presence of Ions 

To analyze the effects of ionic strength on the heat stability (heat-induced aggregation), various concentrations of NaCl solutions were introduced into deamidated protein solutions before subjecting them to heat treatment at 80 °C for 20 min. Specifically, the DPPI solution after PG deamidation was placed in an ice-water bath. Three times the volume of NaCl solution (with varying concentrations and dissolved in 50 mM PBS at pH 7.0) was added to achieve a 5 mg/mL DPPI solution with ionic strengths ranging from 0 mol/L to 1 mol/L. The resulting solution samples were kept in an ice-bath condition. The PPI and HPPI control samples, without undergoing an 80 °C treatment, were also exposed to the same sodium ion concentration and then placed in ice bath. The above protein solutions without subjection to 80 °C, including the DPPI, HPPI and PPI samples, were labeled as iD10, iD15, iD20, iD25, iHPPI, and iPPI, respectively. In addition, the heat-treated counterparts were prepared through subjecting them to heat treatment at 80 °C for 20 min instead of being kept in ice bath and were correspondingly labeled as hD10, hD15, hD20, hD25, hHPPI and hPPI, respectively. The turbidity (A_600_) of the solution samples (containing 0–1 mol/L NaCl) after heat treatment was measured using an Infinite M200pro microplate reader (Tecan, Switzerland). The solution samples with 0 and 0.1 M NaCl were further subjected to molecular weight distribution analysis.

#### 2.6.2. High-Performance Size Exclusion Chromatography (HPSEC) for DPPI Solution Samples with 0 and 0.1 M NaCl

The molecular weight (Mw) distribution of pea protein samples with different DDs (10–25%) with and without heating (80 °C, 20 min) in the presence of 0 and 0.1 mol/L NaCl was determined via high-performance size exclusion chromatography with an Agilent 1260 system equipped with a TSK G3000SWXL column (TOSOH, Tokyo, Japan). The mobile phase was PBS (pH 7.0, 50 mmol/L) containing 0.15 mol/L NaCl at a flow rate of 0.425 mL/min. After filtration through a 0.45 µm filter, 20 µL of the sample (5 mg/mL) was injected into the column, and the absorbance was monitored at 280 nm.

#### 2.6.3. Transmission Electron Microscopy (TEM)

The morphology of the DPPI solution samples after heating at 80 °C for 20 min was characterized with transmission electron microscopy (HT7700, Hitachi Ltd., Tokyo, Japan). The protein solution was first diluted to 0.02% (*w*/*v*) with ultrapure water, and then the diluted sample was dropped on a carbon-coated copper grid for 15 min at room temperature. After removing excess liquid with filter paper, the sample was stained with 1% (*w*/*v*) phosphotungstic acid for 10 min, and then the excess dye was removed. The samples were observed and recorded at an acceleration voltage of 80 kV.

### 2.7. Statistical Analysis

All experiments were conducted in triplicate, except for HPSEC, TEM, and SDS-PAGE. Data were analyzed using SPSS statistical software (version 20.0) and compared via one-way ANOVA at a significance level of 5% (*p* < 0.05).

## 3. Results and Discussion

### 3.1. Structural Characterization

#### 3.1.1. Deamidation Degree, Hydrolysis Degree, and Electrophoretic Analysis of DPPI Samples

**Figure 1:**
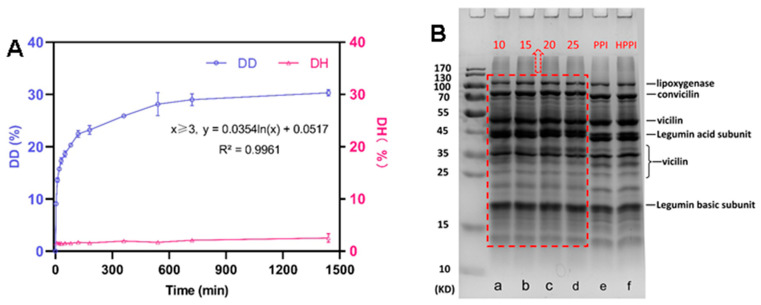
(**A**) Degree of deamidation (DD, %) and degree of hydrolysis (DH, %) as a function of reaction time. (**B**) SDS-PAGE patterns of deamidated pea protein isolates (DPPI) and controls: (a–d in the dotted red box) DPPI with DD of 10%, 15%, 20% and 25%, respectively; (e) PPI; (f) HPPI: heat control.

The deamidation degree (DD) of proteins plays a pivotal role in shaping their structural changes and influencing their functional properties. It is essential to elucidate the reaction time for deamidating pea proteins with different deamidation degree. Figure 1A illustrates the time-dependent evolution of DD in PPI during PG-mediated reactions. Initially, the DD of PPI significantly increased and reached to 22% within the first 2 h. Subsequently, the raising rate slowed down, and it reached a plateau at approximately 28% around 540 min. This observed trend aligns with similar tendencies reported in PG-mediated deamidation of soybean protein [11], wheat gluten [10], and coconut protein [16]. At the end of the 24 h period, the DD of pea protein samples reached approximately 30.3%. The slowdown in the enzymatic reaction during the later stages can be attributed to several factors. First, it may result from a reduction in the amount of substrate glutamine residues within the protein. Additionally, the deceleration in the reaction rate may also be attributed to the denaturation of the PG during the incubation process [17]. Furthermore, the product NH_3_ in the solution (ammonium ions) could act as a competitive inhibitor, reducing the reactivity of glutamine residues [18]. The progress curve of a typical enzyme reaction comprises both linear and nonlinear segments. By fitting the progression curve using nonlinear regression, a logarithmic function (y = 0.0354ln(x) + 0.0517, x ≥ 3, r^2^ = 0.996) was obtained. Accordingly, deamidated PPI samples with four DDs (10%, 15%, 20%, and 25%) required reaction times of 0.91 min, 16.07 min, 65.97 min, and 270.86 min, respectively. These four DDs of deamidated PPI samples were prepared for further investigated to examine their protein properties.

To assess the extent of protein chain fragmentation during PG-mediated deamidation, we measured the proteolysis degree of the DPPI samples (Figure 1A). Notably, the hydrolysis degree value of the DPPI samples remained below 3% throughout the 24 h deamidation period, indicating minimal proteolysis. It is worth noting that the heat control also exhibited a similar DH value of 2.17% compared with the DPPI samples, and this low DH could be attributed to the enzyme inactivation process at 80 °C for 20 min. The high temperature may induce protein unfolding and the release of partial short peptide subunits and proteins, resulting in a slight increase in DH [19]. Thus, we hypothesize that the DH observed in the DPPI samples primarily stems from the enzyme inactivation process at 80 °C. In essence, PG-mediated deamidation has a negligible impact on the hydrolysis of pea protein. Among the DPPI samples, those with four different DDs (10%, 15%, 20%, and 25%) were subjected to SDS-PAGE characterization to further investigate the protein degradation during the deamidation process. The similar electrophoretic bands between the DPPI samples and the PPI samples (Figure 1B) reveal minimal changes in protein chain length after deamidation.

#### 3.1.2. Zeta Potential Analysis

**Figure 2:**
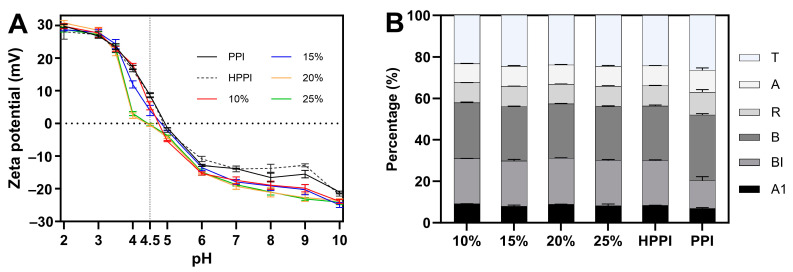
(**A**) Zeta potential profiles of deamidated PPI samples and PPI controls as a function of pH. (**B**) Secondary structure composition of deamidated PPI with DD ranging from 10 to 25% and controls (PPI and HPPI).

Electrostatic attractive and repulsive forces between proteins play a significant role in their solubility [20]. Zeta potential analysis is useful for characterizing electrostatic interactions between dispersed particles and estimating the isoelectric point (pI) [21]. In Figure 2A, the zeta potential of the DPPI samples is plotted as a function of pH. When compared to the PPI and HPPI samples, the zeta potential curve for the DPPI samples exhibited a noticeable shift in their isoelectric point (pI) to lower pH values. This shift was more pronounced in the DPPI samples with 20% and 25% DD compared to those with lower DD (10% and 15%). DPPI samples with lower DD (10% and 15%) exhibited lower pI values than controls but still remained in the pH range of 4.5–5, whereas DPPI with high DD exhibited a significantly lower pI located at pH 4–4.5. In addition to the shift in pI, the DPPI samples exhibited a lower zeta potential magnitude than the HPPI and PPI controls, indicating that the DPPI samples have a higher absolute zeta potential value at pH levels above their pI. DPPI samples with higher DD showed higher absolute zeta potential than those with low DD. These results could be attributed to the increased carboxyl group content resulting from deamidation [22], which increases the number of ionizable groups on the surface of pea protein. However, it is noteworthy that the further increase in carboxyl groups did not significantly alter the negative charge density on the protein surface, as supported by the overlapping zeta potential curves for the DPPI samples with 20% and 25% DD. Therefore, it can be concluded that a 20% DD can significantly change the negative charge density on the pea protein surface.

#### 3.1.3. Secondary Structure Analysis

The amide I region (1600–1700 cm^−1^) in the FTIR spectra is a sensitive region for analyzing the secondary structures of proteins. Gaussian curve fitting of the amide I region (Figure S1) showed four main Gaussian bands at 1630–1638 cm^−1^, 1640–1650 cm^−1^, 1650–1660 cm^−1^ and 1660–1680 cm^−1^. These four bands corresponded to β-sheet (B), random coil (R), α-helix (A) and β-turn (T), respectively. Additionally, the observed weak bands in the ranges of 1610–1615 cm^−1^ and 1690–1695 cm^−1^ (A1) might be attributed to protein amino acid side chains [23]. The bands in the ranges of both 1618–1623 cm^−1^ and 1678–1682 cm^−1^ (BI) were reported to result from the absorption of intermolecular β-sheets, which is a characteristic of aggregation [24].

The secondary structure changes in the PPI samples after deamidation are presented in Figure 2B. PPI samples consisted of 31.7% β-sheet (B), 10.6% α-helix (A), 11.0% random coil (R), 26.5% β-turn (T) and 13.4% intermolecular β-sheet (BI), which aligns with the findings from a previous report [25]. DPPI and HPPI samples showed a reduction in the β-sheets (B) content and a simultaneous increase in the intermolecular β-sheet (BI) content compared to the PPI samples. However, negligible differences were observed between the DPPI and HPPI samples. This result could be attributed to the formation of heat-induced intermolecular β-sheet aggregates during the 80 °C treatment rather than the deamidation effect [26]. These results suggest that deamidation would hardly alter the secondary structure of pea proteins, which is consistent with previous findings regarding PG-treated α-lactalbumin [18].

#### 3.1.4. Tertiary Structure Analysis

**Figure 3:**
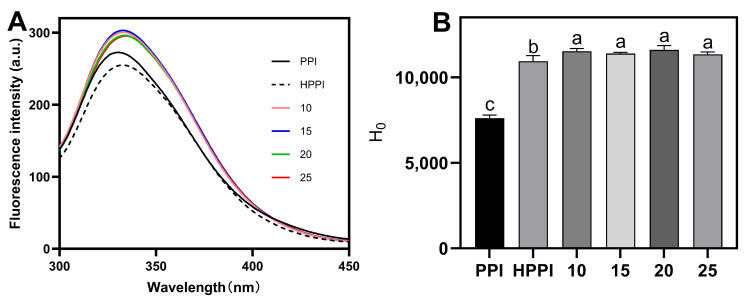
Fluorescence spectra (**A**) and surface hydrophobicity (**B**) of deamidated PPI (with different DDs) and controls (PPI and HPPI). Different lowercase letters in (**B**) indicate significant differences (*p* < 0.05).

The intrinsic fluorescence of proteins in solution is commonly used to detect the conformational changes. Upon excitation at 290 nm, the fluorescence signal primarily arises from tryptophan residues, and the fluorescence peak is significantly influenced by the microenvironmental polarity surrounding these aromatic amino acid residues. As shown in Figure 3A, the PPI samples displayed lowest λ_max_ at 330 nm. However, the HPPI samples exhibited a redshift to 333.07 nm along with a decrease in fluorescence intensity. This redshift could be attributed to protein unfolding induced by the heat treatment, which alters the environment surrounding the Trp residues, making it more polar. The decrease in intensity may result from the combined effects of protein unfolding and subsequent aggregation during heat treatment, which is in line with previous findings in heat-treated milk proteins at 90 °C [27]. In contrast, the DPPI samples exhibited redshifts along with increased fluorescence intensity compared to the PPI samples. Low-DD (10 and 15%) DPPI samples exhibited a λ_max_ at 333.07 nm, while high-DD (20 and 25%) DPPI showed a higher λ_max_ at 334 nm. The more pronounced redshift in high-DD DPPI compared to HPPI indicated a more extended protein structure, which can be attributed to the increased electrostatic repulsion between carboxyl groups following deamidation [28]. The accompanying increase in fluorescence intensity in the DPPI samples compared to the HPPI samples could be due to the deamidation-induced enhancement of electrostatic repulsion (as supported by the zeta potential results). This enhancement hindered protein aggregation through disulfide bonds and hydrophobic interactions during heating, allowing for more hydrophobic groups to remain exposed. [29,30].

The surface hydrophobicity results revealed similar conformational changes upon deamidation. As shown in Figure 3B, the heat control samples exhibited higher *H*_0_ compared to the PPI control, attributed to the heat-induced exposure of hydrophobic groups. In contrast, DPPI showed higher (*p* < 0.05) surface hydrophobicity than the PPI and HPPI samples. This observation suggests that PG-mediated deamidation exposed partially hydrophobic structural domains of PPI [8], consistent with previous findings regarding PG-treated whey protein isolates [22]. These results collectively demonstrate that PPI structures unfold after PG-mediated deamidation, and DPPI with high DD exhibited even greater unfolding.

### 3.2. Solubility Analysis

**Figure 4:**
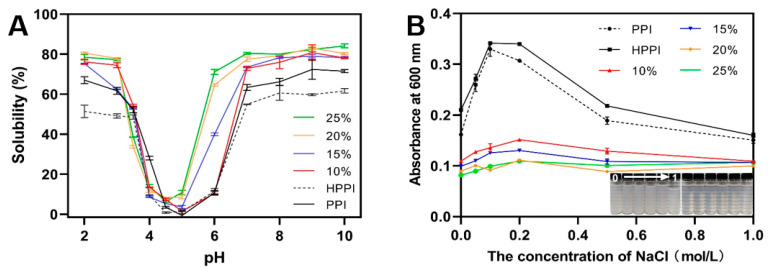
(**A**) Solubility profile of deamidated PPI as a function of pH. (**B**) Changes in turbidity of deamidated PPI solutions after heat treatment at 80 °C at the concentration of 0–1 mol/L NaCl. The corresponding appearances of HPPI control (left image) and deamidated PPI with a DD of 25% (right image) after heating at 0–1 mol/L NaCl are shown in the inset images.

The solubility of the PPI, DPPI and HPPI samples in the pH range of 2.0 to 10.0 is presented in Figure 4A. Typical U-shaped solubility-pH profiles were observed for all protein samples. The HPPI samples exhibited the lowest solubility across a wide pH range (2.0–10.0). The PPI control showed a comparable solubility curve but higher solubility at specific acidic and alkaline pH values (2, 3, and 7–10). In contrast, the solubility profile of the DPPI samples exhibited an upward shift, indicating that the deamidation treatment improved the solubility of DPPI. Furthermore, the extent of the protein solubility curve shifting upward with the DD, suggesting that protein solubility increased along with DD. However, when the DD reached 20% or higher, the solubility curve showed little to no significant change. The solubility curves of proteins at 20% DD and 25% DD almost overlap. The solubility at pH 7, a commonly used metric for assessing protein solubility, demonstrated significant improvements for the DPPI samples. Specifically, the solubility at pH 7 for DPPI with a DD of 10%, 15%, 20%, and 25% was measured at 72.9%, 73.6%, 77.5%, and 80.4%, respectively. These values were significantly higher than the solubility of PPI (63.4%) and HPPI (55.0%) samples. Additionally, the accompanying leftward shift in the solubility curve indicates that the pI of the DPPI samples moved to a lower pH, and this shift was more pronounced with the increase in DD. High-DD DPPI (20% and 25%) showed their lowest solubility at around pH 4.5, while low-DD DPPI exhibited this at approximately pH 5.0. These results indicate that significant changes occurred in the electrostatic properties of pea protein when the DD reached around 20%. The increased solubility of the DPPI samples and the shift in the isoelectric point toward acidity after PG modification would be attributed to the deamidation-induced conversion of protein amide groups into carboxyl groups. This increase in charge leads to unfolding conformation of the proteins, enhancing interactions between the protein and water and resulting in improving solubility [31]. Additionally, the increase in carboxyl groups would increase the number of ionizable amino acid residues in pea protein, thus lowering its isoelectric point.

### 3.3. The Heat Properties (Resistance against Thermal Aggregation) of DPPI

#### 3.3.1. The Turbidity of DPPI Solutions with Different NaCl Concentrations after Heat Treatment

Measuring turbidity is a fundamental method for monitoring protein aggregation due to the scattering potential of protein aggregates [32]. Figure 4B illustrates the turbidity changes in DPPI following heat treatment at increasing NaCl concentrations ranging from 0 mol/L to 1 mol/L. Specially, the PPI samples exhibited a turbidity curve similar to that of the HPPI samples, albeit with a slightly lower magnitude. The HPPI samples exhibited the highest turbidity than the PPI and DPPI samples. In contrast, the DPPI samples showed a significant decrease in turbidity compared to the PPI and HPPI samples, with high-DD DPPI exhibiting lower turbidity compared to low-DD DPPI samples. Notably, the turbidity curve of the DPPI with a 20% DD closely overlaps with that of the DPPI with a 25% DD. These results can be attributed to the deamidation-induced enhanced electrostatic repulsion between protein molecules, as supported by the zeta potential findings. The conversion of protein amide groups to carboxyl groups impedes the aggregation of unfolded proteins during heat treatment, leading to a reduction in turbidity [30].

#### 3.3.2. Molecular Weight Distribution of DPPI Solutions with and without Added 0.1 mol/L NaCl after Heat Treatment

**Figure 5:**
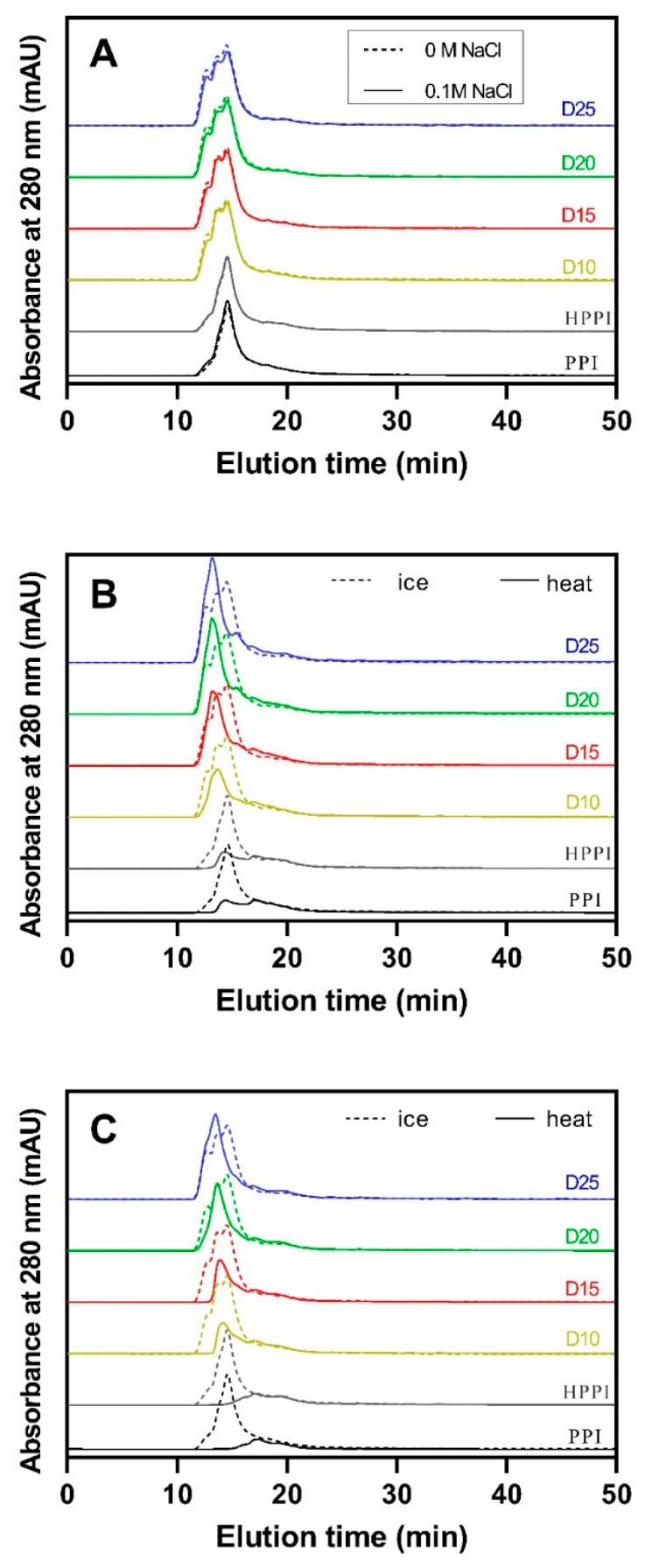
Molecular weight distribution profiles. (**A**) Deamidated PPI (without 80 °C heat treatment) with and without the addition of 0.1 mol/L NaCl. (**B**) Deamidated PPI samples in the absence of NaCl with and without heat treatment at 80 °C for 20 min. (**C**) Deamidated PPI samples with 0.1 mol/L NaCl with and without heat treatment at 80 °C for 20 min. Solid and dashed lines represent the protein samples with and without heating at 80 °C for 20 min, respectively.

Figure 5A depicts the molecular weight distribution of DPPI before and after the addition of 0.1 mol/L NaCl, without undergoing an 80 °C heat treatment. Compared to the iPPI control and iHPPI control samples, the DPPI samples (iD10–iD25) exhibited a molecular weight distribution graph with a different peak shape, although their peak positions remained nearly unchanged. This result is likely due to changes in protein conformation rather than molecular weight (as supported by the results of SDS-PAGE). The slight decrease in elution time of the DPPI samples could be attributed to an increase in the apparent molecular size resulting from deamidation-induced protein unfolding [22].

Figure 5B shows the molecular weight distribution profiles of the DPPI samples in the absence of salt ions before and after heating step at 80 °C for 20 min. Both the PPI and HPPI control samples exhibited reduced peak areas after heating compared to their pre-heating states. This decrease could be attributed to the formation of protein aggregates during heating. However, these protein aggregates were effectively removed using a 0.45 micrometer membrane during sample preparation for HPLC. Globular proteins would unfold during heating, exposing active groups and promoting extensive protein aggregation, leading to the formation of insoluble protein aggregate precipitates [30]. In contrast, the peak areas of the DPPI samples did not decrease after heating and exhibited similar or even stronger peak intensities compared to before heating, especially in samples with 15%, 20%, and 25% DD. Moreover, the peak intensities of the DPPI samples after heating surpassed those of the PPI and HPPI samples. This observation may be attributed to the increased electrostatic repulsion after deamidation, which hindered protein aggregation in the heat-unfolded protein. The resulting exposure of aromatic amino acid residues likely contributed to stronger absorption, enhancing the signal at 280 nm [33]. However, DPPI with 10% DD exhibited a slight decrease in the peak area after heating. This reduction may be attributed to the limited increase in charges, as indicated by the zeta potential results, which could not effectively prevent extensive protein aggregation of the heat-unfolded protein.

Figure 5C demonstrates the molecular weight distribution profiles of the DPPI samples with the added 0.1 mol/L NaCl before and after heating. The absorption peaks of the PPI control and HPPI control in 0.1 mol/L NaCl almost disappeared after heating. In contrast, this reduction in peak area after heating was mitigated in the DPPI samples, especially in the high-DD DPPI samples. The obvious decrease in peak area in the low-DD (10% and 15% DD) DPPI samples were likely because their slight increase in negative charge was insufficient to prevent protein aggregation caused by ion screening at 0.1 mol/L NaCl. The added ions screened the charged protein chains, reducing the electrostatic repulsion between protein molecules and facilitating protein aggregation [34]. These results indicate that deamidation at a high level (above 20%) effectively prevents the extensive thermal aggregation of pea proteins, regardless of the presence of ions.

#### 3.3.3. Morphology of the DPPI Samples after Heating at 80 °C for 20 min

**Figure 6:**
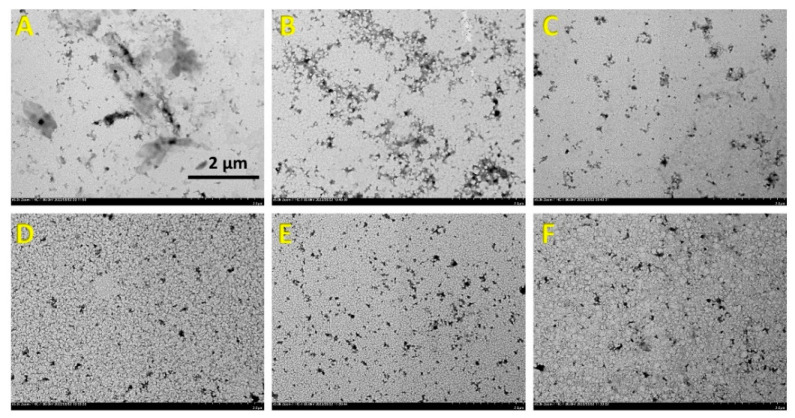
Morphology (TEM images, 5000×) of deamidated PPI, PPI and HPPI control after heating at 80 °C for 20 min. (**A**) PPI; (**B**) HPPI; (**C**–**F**) deamidated PPI with DD of 10%, 15%, 20% and 25%, respectively.

TEM was employed to examine the morphology of pea protein aggregates formed after exposure to 80 °C heat treatment. As shown in Figure 6, both the PPI and HPPI samples exhibited densely packed, amorphous aggregates with a couple of micrometers in size after the heating treatment (Figure 6A,B), which is typical for globulins [35]. It is worth noting that these aggregates were filtered out during sample preparation for HPSEC and therefore did not appear in the molecular weight distribution profiles. In contrast, the amount of protein aggregates in the DPPI samples decreased after heating and their particle size became smaller, exhibiting a more uniform distribution (Figure 6C–F). Notably, DPPI with 10% DD still formed protein aggregates, albeit with noticeably reduced sizes (Figure 6C). These findings, indicating limited protein aggregation in the DPPI samples, align well with the HPSEC results showing either weak or no reduction in the peak compared to the control. The reduced protein aggregation of DPPI could be ascribed to enhanced electrostatic repulsion, which outweighs the increased hydrophobic attraction resulting from deamidation-induced unfolding and heat treatment [35].

### 3.4. A Supposed Mechanism for the Improvement in Solubility and Heat Properties after Deamidation

**Figure 7:**
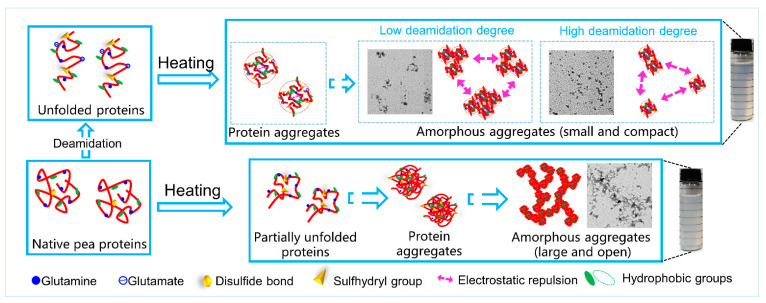
Schematic depiction of the mechanism for improvement in solubility and heat stability of PPI after deamidation.

Figure 7 illustrates the mechanism behind the enhanced solubility and heat stability (resistance against thermal aggregation) of pea proteins induced by deamidation. When subjected to deamidation with PG, partial amide groups of PPI is converted into carboxyl groups, leading to an increase in negative charges. The enhanced electrostatic repulsion causes protein molecular structure to unfold, exposing more charged and polar groups to the surrounding water. This structural change and the increase in charged patches would promote interactions between pea proteins and water, thereby improve solubility.

Upon heating at 80 °C, the PPI samples, primarily consisting of globular proteins, would undergo thermal unfolding process. The exposed active groups would result in extensive protein aggregation, forming large and insoluble protein aggregates. In contrast, the increase in charged carboxyl groups in the DPPI samples would hinder protein–protein interactions due to the strong electrostatic repulsion. This, in turn, prevents the extensive protein aggregation during heat treatment. In the absence of salt ions, the mild increase in charge in the DPPI samples with low DD (10%) could not effectively prevent the protein interactions, thus resulting in limited protein aggregations. However, in the presence of 0.1 mol/L NaCl, the increased charge in the low-DD DPPI samples (both DD 10% and 15%) is screened, resulting in protein aggregation. It is worthy to note that DPPI samples with high DDs (20% and 25%) inhibit the heat-induced aggregation regardless of the presence of ions. This inhibition could be attributed to the retention of the residual charges even after screening by ions. The consistent trends observed in zeta potential, intrinsic fluorescence, solubility, turbidity, and molecular weight distribution properties indicate that a DD of around 20% can lead to significant alternations in pea protein structure and result in substantial changes in their functional properties.

## 4. Conclusions

Pea proteins were isolated from raw pea seeds and subsequently subjected to deamidation using PG enzyme, resulting in deamidated pea protein isolates (DPPIs) with varying degrees of deamidation (DD, 10–25%). The deamidation process significantly improved the solubility and heat stability of the DPPI samples when compared to the unmodified pea protein isolates (PPIs), with the most substantial enhancements observed in the DPPI samples with higher DDs (20% and 25%). The transformation of amide groups into carboxyl groups during deamidation induced a certain degree of protein unfolding, exposing more hydrophobic regions, while the secondary structure remained unchanged. Consequently, the enhanced solubility and heat stability can be attributed to the heightened electrostatic repulsion between carboxyl groups. This repulsion promotes protein unfolding, facilitates protein–water interactions, and inhibits the aggregation of unfolded proteins in the presence of salts at elevated temperatures. These characteristics suggest that deamidated PPI holds promise as a plant-derived protein ingredient suitable for liquid formulas, such as drink and dairy milk substitute. However, further research is imperative to explore additional functional properties, such as their emulsifying and foaming characteristics, to expand the applications of deamidated PPI within the food industry.

## Data Availability

The data presented in this study are available on request from the corresponding author.

## Supplementary Materials

The following supporting information can be downloaded at: https://www.mdpi.com/article/10.3390/foods12224130/s1, Figure S1: Original (dotted line) and curve fitting spectra (solid line) of amide I of FTIR for pea protein isolates (PPI) samples (Samples Corresponding to Each Figure Indicated in upper right corner: NPPI, unmodified PPI; HPPI, heat PPI control; D10 ~ D25, deamidated PPI with degree of deamidation Ranging from 10% to 25%).
