# Supplementary material for: Enhancing Solubility and Reducing Thermal Aggregation in Pea Proteins through Protein Glutaminase-Mediated Deamidation
Source: Foods. 2023 Nov 15;12(22):4130. doi: 10.3390/foods12224130 (PMC10670925; doi:10.3390/foods12224130)

Figure S1. Original (dotted line) and curve fitting spectra (solid line) of amide I of FTIR for pea protein isolates (PPI) samples (Samples Corresponding to Each Figure Indicated in upper right corner: NPPI, unmodified PPI; HPPI, heat PPI control; D10 ~ D25, deamidated PPI with degree of deamidation Ranging from 10% to 25%)

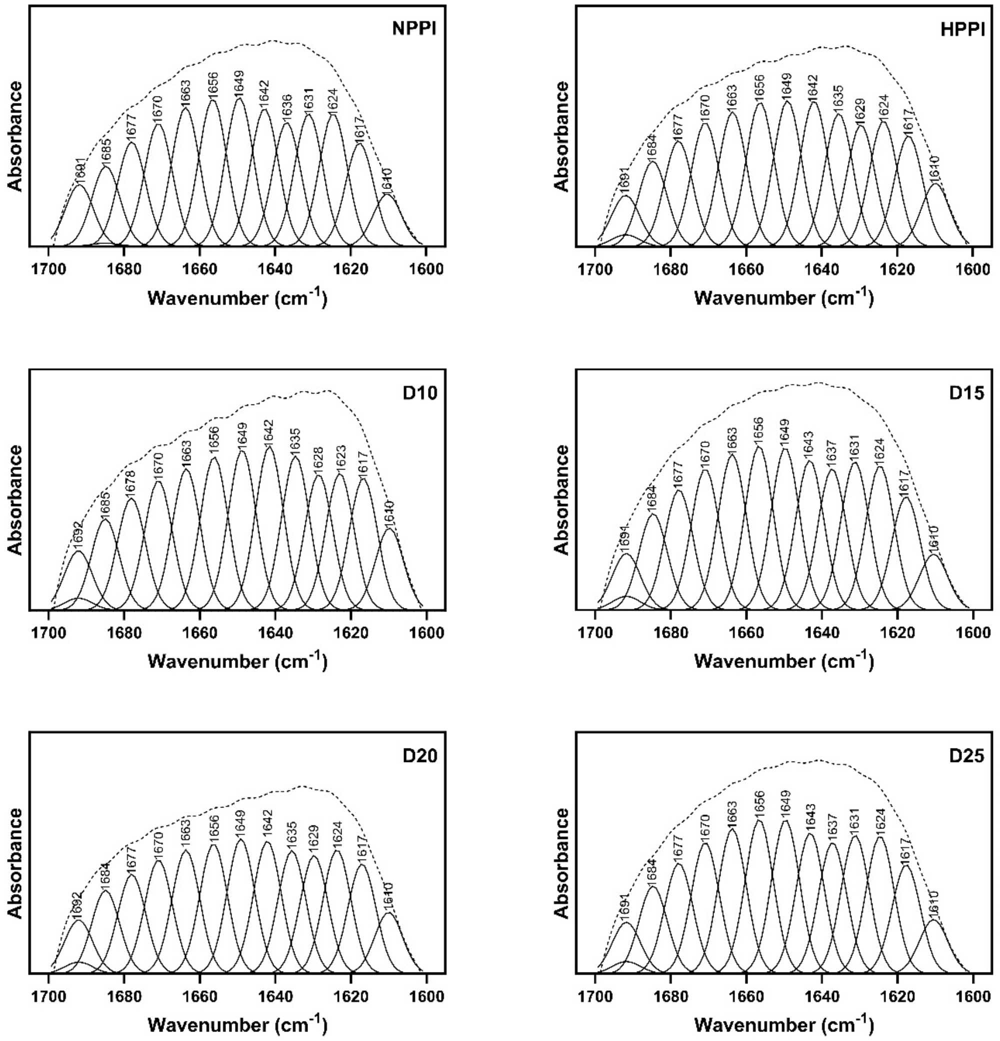

Supplement: Supplementary file 1 [file foods-12-04130-s001.zip › foods-2700655-supplementary.pdf]
